# Gene expression profiles of patients with cerebral hematoma following spontaneous intracerebral hemorrhage

**DOI:** 10.3892/mmr.2014.2421

**Published:** 2014-07-25

**Authors:** TAO YANG, JIANWEN GU, BIN KONG, YONGQIN KUANG, LIN CHENG, JINGMIN CHENG, XUN XIA, YUAN MA, JUNHAI ZHANG

**Affiliations:** 1 Department of Neurosurgery, Chengdu Military General Hospital, Chengdu, Sichuan 610083, P.R. China; 2 Department of Postgraduate, Third Military Medical University, Chongqing 400038, P.R. China

**Keywords:** spontaneous intracerebral hemorrhage, perihematomal tissue, differentially expressed gene, interaction network, function and pathway enrichment analysis

## Abstract

The present study aimed to investigate the gene functions and expression profiles in perihematomal (PH) brain regions following spontaneous intracerebral hemorrhage. The gene expression profiles were downloaded from the Gene Expression Omnibus database under accession number GSE24265, which includes 11 brain samples from different regions, including four samples from PH areas, four from contralateral grey matter (CG) and three from contralateral white matter (CW). The gene expression profiles were pre-processed and the differentially expressed genes (DEGs) between PH and CG tissue, and PH and CW tissue were identified using R packages. The expression of genes in different tissues was analyzed by hierarchical clustering. Then, the interaction network between the DEGs was constructed using String software. Finally, Gene Ontology was performed and pathway analysis was conducted using FuncAssociate and Expression Analysis Systematic Explorer to identify the gene function. As a result, 399 DEGs were obtained between PH and CG, and 756 DEGs were identified between PH and CW. There were 35 common DEGs between the two groups. These DEGs may be involved in PH edema by regulating the calcium signaling pathway [calcium channel, voltage-dependent, T-type, α1I subunit, Ca^2+^/calmodulin-dependent protein kinase II α (CAMK2A), ryanodine receptor 2 (RYR2) and inositol 1,4,5-trisphosphate receptor, type 1 (ITPR1)], cell proliferation (sphingosine kinase 1), neuron differentiation (Ephrin-A5) or extracellular matrix-receptor interaction [collagen, type I, α 2, laminin B1 (LAMB1), syndecan 2, fibronectin 1 and integrin α5 (ITGA5)]. A number of genes may cooperate to participate in the same pathway, such as ITPR1-RYR2, CAMK2A-RYR2 and ITGA5-LAMB1 interaction pairs. The present study provides several potential targets to decrease hematoma expansion and alleviate neuronal cell death following spontaneous intracerebral hemorrhage.

## Introduction

Spontaneous intracerebral hemorrhage (ICH) involves the extravasation of blood within the brain parenchyma in the absence of trauma or surgery, accounting for 10–30% of all strokes worldwide (1). ICH is a complex, dynamic process that consists of three distinct phases (2): i) Initial hemorrhage, retraction of the clot begins in the first few hours following ICH. ii) Hematoma expansion, hematoma expansion occurs 24 or 48 h following the initial onset of symptoms, which involves a sudden increase in intracranial pressure, disrupting the integrity of the local tissue and blood-brain barrier. In addition, obstructed venous outflow promotes the activation of tissue thromboplastin, resulting in local coagulopathy. (iii) Perihematoma (PH) edema, PH cerebral edema develops within the first few days following ICH as a result of an inflammatory response [activation of cellular (leukocytes, macrophages and microglia) and molecular (cytokines, proteases and reactive oxygen species) components] (3) secondary to local release of thrombin and other end products of coagulation from the hematoma, and also due to cytotoxic mediators (hemoglobin and its degradation products, heme and iron) that are released from red blood cells (4–6).

The hematoma expansion rate and PH edema volume are key factors to predict neurological deterioration and survival. Hematoma volume >30 ml is associated with increased mortality (7). When the hemorrhagic volume exceeds 150 ml, cerebral perfusion pressure falls to zero, which subsequently leads to patient mortality (8). Therefore, prevention of hematoma expansion is a logical therapeutic target (9). Additionally, it is possible that anti-inflammatory and anti-apoptotic drugs may have the therapeutic potential to ameliorate secondary brain injury following ICH. For example, sesamin confers neuroprotection in rat ICH by suppressing activation of microglia and the p44/42 MAPK pathway (10). Ginkgolide B was able to significantly suppress the gene expression of Toll-like receptor 4 and nuclear factor-κB, reduce the concentrations of tumor necrosis factor-α, interleukin (IL)-1β and IL-6 as well as reduce the number of apoptotic neuronal cells in hemorrhagic rat brain tissues (11).

Recently, Carmichael *et al* (12) attempted to investigate the gene expression pattern in PH tissues of ICH rat models in a microarray study, which simultaneously measured transcript abundances of thousands of genes in a cell population or tissue. The results indicated a robust upregulation of pro-/anti-inflammatory networks and a downregulation of neuronal signaling pathways (12). Furthermore, Rosell *et al* (13) investigated the genomic profile following spontaneous human ICH and suggested that the significantly overexpressed genes in the PH areas encoded for cytokines, chemokines, coagulation, cell growth and proliferation factors, while the downregulated genes encoded proteins involved in the cell cycle or neurotrophins. Therefore, the identified genes may be potential targets for ICH therapy.

The present study aimed to further identify the differentially expressed genes (DEGs) between PH and contralateral healthy tissues [grey (CG) matter and white (CW) matter] using a different statistical approach and threshold. Further interaction network and function analyses were also performed for these DEGs. It is expected that these data may further elucidate the processes and mechanisms of death caused by hematoma following spontaneous ICH.

## Materials and methods

### Microarray data

The gene expression profiles were downloaded from the Gene Expression Omnibus under accession number GSE24265 (13), which includes 11 brain samples from different regions of four deceased patients who had suffered from a supratentorial ICH within four days prior to mortality. Of these samples, four were from PH tissues, four from CG and three from CW. The study group included two female and two male subjects with a median age of 79 years (range, 68–92 years). The samples from PH, CG and CW tissue were obtained within the first 5 h following mortality, snap frozen in liquid nitrogen and stored at −80°C until RNA isolation. The original studies by Rosell *et al* (13) was approved by the Ethics Committee of the Vall d’Hebron Hospital (Barcelona, Spain) and informed and written consent was acquired from all of the patients or patient’s relatives. Demographic and tissue sampling data are presented in Table I. The array platform was GPL570 [Human Genome U133 Plus 2.0 Array (Affymetrix Inc., Santa Clara, CA, USA)]. The array annotation information was from Affymatrix, including the information for all probes on Affymetrix ATH1 (25K; (Affymetrix Inc.).

### Data pre-processing and identifying DEGs

Firstly, the raw data were transformed into a readable expression profile format and then the missing data were imputed according to Troyanskaya’s method (14) followed by normalization (15). DEGs between PH and CG, and PH and CW were identified using the limma package in R (16). Benjamini-Hochberg (BH) multiple test correction was then used to adjust the P-values (17). Only the genes with a false discovery rate (FDR) <0.05 and |logFC(fold change)| >1 were selected as DEGs.

### Tissue specific DEGs

The expression of genes is time- and space-specific, and in different tissues may be significantly different. The genes were clustered based on their expression in two groups using Cluster analysis (18). The expression differences in the various tissues were visualized by heatmap (Clifton Watt, Stanford University, USA).

### Construction of the interaction network

Numerous activities in the body, including important physiological activities and responses to the external or internal environment, are performed by the binding and dissociation of proteins. All of these activities are conducted by signaling transduction networks consisting of protein-protein interactions (19). Therefore, detailed investigations into protein interaction networks is essential for the understanding of bodily systems (20). Based on the sequence and structure information of DEGs, their interaction possibility was predicted according to the String database (http://string.embl.de/) that quantitatively integrates interaction data from four sources [genomic context, high-throughput experiments, (conserved) co-expression and previous knowledge] for a large number of organisms, and transfers information between these organisms where applicable (21). Furthermore, the interaction network was also constructed for the two groups; PH vs. CG and PH vs. CW.

### Functional enrichment of DEGs in interaction networks

The annotation was performed for the genes in protein interaction networks. FuncAssociate (http://llama.med.harvard.edu/funcassociate) is a web application that identifies properties enriched in lists of genes or proteins that emerge from large-scale experimentation (22). The interactive DEGs were screened and functional enrichment analysis was performed using FuncAssociate (22). FDR<0.05 was set as the threshold for this analysis using the hypergeometric distribution.

### Pathway analysis of DEGs in interaction networks

Expression Analysis Systematic Explorer (EASE, http://david.niaid.nih.gov/david/ease.htm) is a customizable, standalone software application that facilitates the biological interpretation of gene lists derived from the results of microarray, proteomic and Serial Analysis of Gene Expression studies (23). EASE queries the Kyoto Encyclopedia of Genes and Genomes pathways database (http://www.genome.ad.jp/kegg/) and determines functional enrichment by calculating a Fisher’s exact test P-value for each pathway. P≤0.05 was considered to indicate as significant gene-enrichment in a specific annotation category.

## Results

### Screening of DEGs

**Figure 1:**
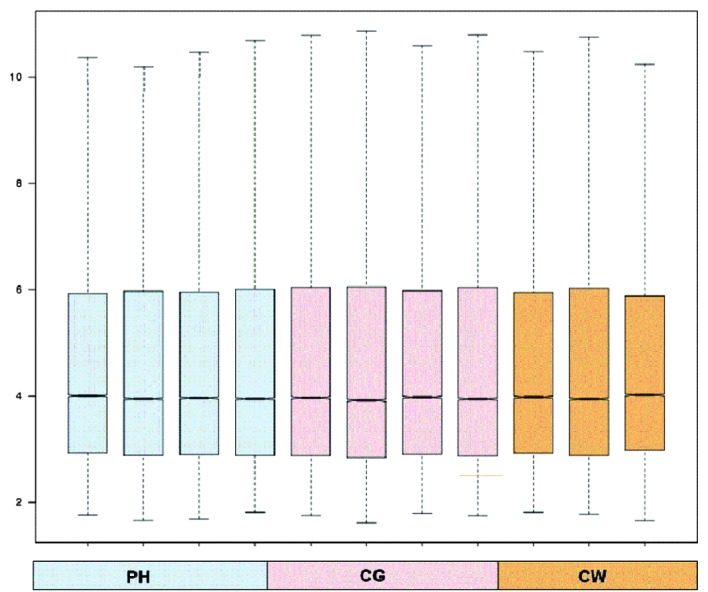
Box plots of normalized gene expression data. Blue, pink, orange, respectively represents brain PH, CG and CW. The median of normalized data are almost at the same level, indicating good standardization. PH, perihematomal tissue; CG, contralateral gray matter; CW, contralateral white matter.

**Figure 2:**
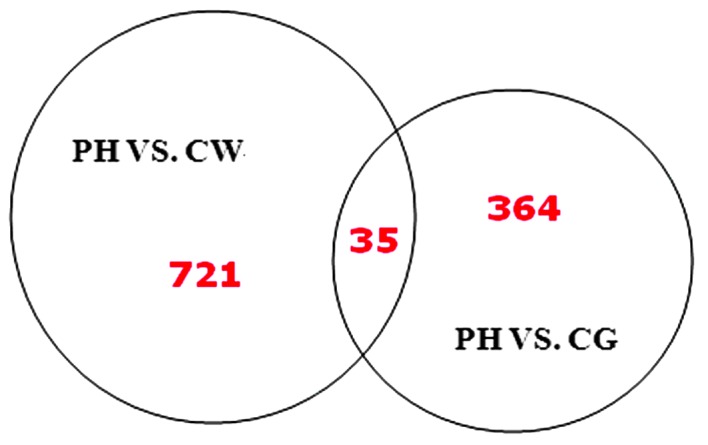
DEGs of the two groups. The two circles represent the DEG set of PH vs. CW and PH vs. CG. The red numbers indicate number of DEGs. PH, perihematomal tissue; CG, contralateral gray matter; CW, contralateral white matter; DEGs, differentially expressed genes.

**Figure 3:**
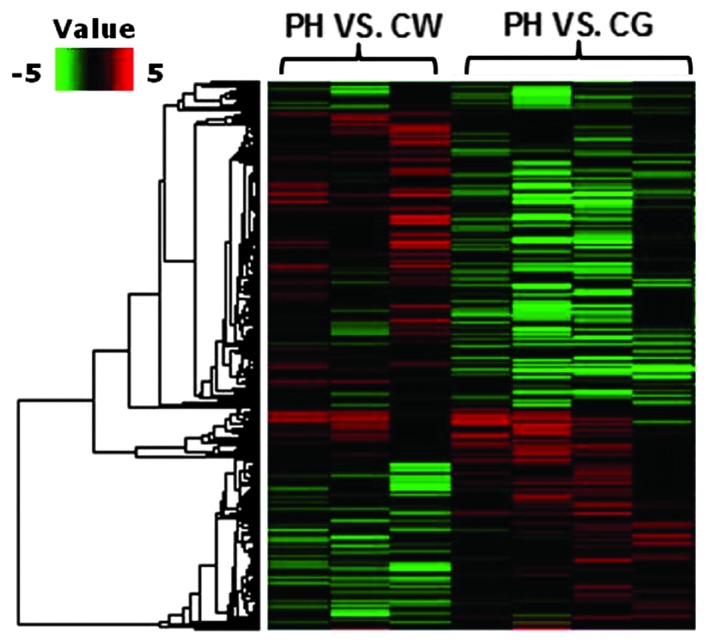
Hierarchical clustering of gene expression. Red represents over-expression and green represents low expression. The left shows PH vs. CW and the right PH vs. CG. PH, perihematomal tissue; CG, contralateral gray matter; CW, contralateral white matter.

Following data pre-processing, the gene expression profile data with higher normalization (Fig. 1) were selected for DEG analysis. The DEGs were analyzed using the R language limma package and BH multiple test correction. As a result, 399 DEGs were obtained between PH and CG, and 756 DEGs between PH and CW according to the threshold of FDR<0.05 and |logFC|>1, indicating spatial specificity of the gene expression (Fig. 2). Furthermore, there were 35 common DEGs between the two groups (Fig. 3). Among the common DEGs, the expression change tendency for a number of the genes was similar [sphingosine kinase-1 (SPHK1) was downregulated in PH samples compared with CG and CW]), but for a number was opposite [ephrin-A5 (EFNA5) and HTR2A were significantly upregulated in PH samples compared with CG but downregulated compared with CW].

### Co-expression network

**Figure 4:**
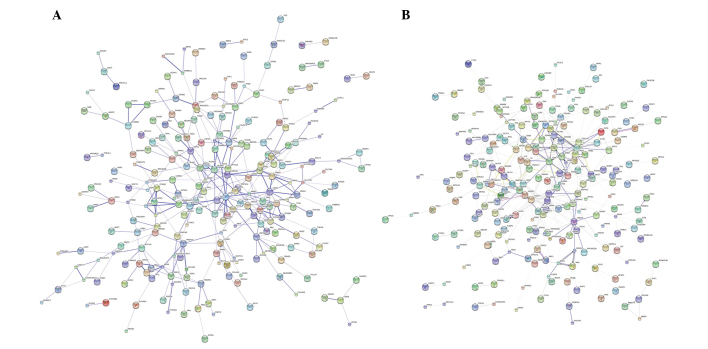
Interaction network of DEGs. (A) The network for PH vs. CG and (B) the network for PH vs. CW. PH, perihematomal tissue; CG, contralateral gray matter; CW, contralateral white matter; DEGs, differentially expressed genes.

The co-expression network for the DEGs between the PH vs. CG and PH vs. CW groups were constructed utilizing String (Fig. 4). The results screened several interactive pairs, including inositol 1,4,5-trisphosphate receptor, type 1 (ITPR1)-ryanodine receptor 2 (RYR2) and Ca^2+^/calmodulin-dependent protein kinase II α (CAMK2A)-MAPK1 in the PH vs. CG group, and integrin α5 (ITGA5)-laminin B1 (LAMB1) in the PH vs. CW group.

### Enriched Gene Ontology (GO) terms of genes in the co-expression network

The DEGs in the co-expression network were enriched into GO terms using FuncAssociate. The results indicated that the GO terms enriched by the two groups were different (Table II). Compared with CG, DEGs for PH were significantly enriched in calcium ion transport (GO:0006816), ion transport (GO:0006811) and synaptic transmission (GO:0007268). Whereas compared with CW, the DEGs of PH were significantly associated with blood vessel development (GO:0001568), vasculature development (GO:0001944) and regulation of cell proliferation (GO:0042127).

### Pathway enriched by DEGs in network

Pathway analysis for DEGs of the two groups was also performed using EASE. As demonstrated in Table III, the four pathways were identified to be significantly correlated with PH when compared with CG, including the Ca^2+^ signaling pathway (hsa04020), which was consistent with the GO enrichment analysis (Table II). A number of genes were shared by enriched GO terms and pathways, including SLC8A2, calcium channel, voltage-dependent, T-type, α1I subunit (CACNA1I), ITPR1, PRKCB, RYR2, CACNA1E, PPP3CA and CAMK2A. Six pathways were enriched for DEGs between PH and CW, among which extracellular matrix (ECM)-receptor interaction (hsa04512) was the most significant pathway. ECM-receptor interaction is most commonly associated with cell proliferation and apoptosis. Therefore, the pathway analysis results of the DEGs in PH vs. CW group were also in accordance with the GO pathway [the common genes were collagen type 1, α2 (COL1A2), LAMB1 and ACTN1].

## Discussion

The present study identified >350 genes that were significantly differentially expressed following ICH in PH regions compared with contralateral healthy tissues (grey matter and white matter) in human samples according to the threshold of FDR<0.05 and |logFC|>1. The majority of DEGs were further revealed to be involved in the networks and pathways correlated with the calcium signaling pathway, cell proliferation, regulation of apoptosis, neuron differentiation or ECM-receptor interaction.

As brain metabolism is almost entirely dependent on the oxidation of glucose delivered by the blood, obstructed blood flow during ICH may cause a rapid decline in tissue metabolism that culminates in neuronal cell death. Although multiple factors are involved, disturbances in neuronal ionic homeostasis may be fundamental for neuronal cell swelling and death (24). Dissipation of ionic gradients causes neuronal depolarization and the release of neurotransmitters from intracellular stores, including glutamate. Glutamate provokes further depolarization due to activation of the α-amino-3-hydroxy-5-methyl-4-isoxazolepropionic acid and N-methyl-D-aspartate glutamate receptors, as well as voltage-gated Ca^2+^ channels, thus triggering Ca^2+^ influx and release from extracellular spaces. In addition, Ca^2+^ is mobilized within the cell from intracellular Ca^2+^ stores through two types of Ca^2+^ release channels: Ryanodine receptors (RyRs) and inositol 1,4,5-trisphosphate receptors (IP3Rs) (25,26). Such Ca^2+^ accumulation and excitation contributes to the generation of reactive oxygen and nitrogen species, and the release of apoptotic factors, ultimately causing cell death through excitotoxicity (27). As expected, the present results implied that CACNA1I, CAMK2A, RYR2 and ITPR1 were significantly upregulated in PH areas. There is also evidence that intracellular Ca^2+^ release channels may cooperate, leading to positive feedback during activation (28). Agonist-dependent activation of IP3Rs may promote activation of RyRs, amplifying and shaping the resulting Ca^2+^-signal (28). The ITPR type 2 channel activity is modulated by Ca^2+^/CaMKII-mediated phosphorylation of serine 150 of the IP3R2 (29). As expected, the present study also found an interaction between RYR2 and ITPR1 (Fig. 4).

Consistently several genes that regulate neuronal cell proliferation were downregulated but the pro-apoptotic genes were upregulated in the PH area. For example, SPHK1 is an enzyme that catalyzes the phosphorylation of sphingosine to form sphingosine-1-phosphate (S1P). S1P regulates diverse biological processes including proliferation and survival following binding to S1P receptor-1 to 5 (S1P1 to S1P5) (30,31). Furthermore, translational investigations suggest a profound impact of S1P administration in the modulation of edema formation in disease state in which increased vascular permeability is a hallmark feature (32). Isoflurane delays the development of early brain injury following subarachnoid hemorrhage through upregulating SPHK1 expression (33).

EFNA5 is reported to be upregulated in reactive astrocytes following stroke, which limits axonal sprouting from cortical neurons and motor recovery. Blockade of EFNA5 signaling using a unique tissue delivery system induces the formation of a novel pattern of axonal projections in motor, premotor and prefrontal circuits, and mediates neuronal recovery (34). As expected, EFNA5 was also upregulated in PH regions compared with normal CG, but it was downregulated compared with CW (conveys axons), indicating the ephrin/Eph A expression gradients in the central nervous system (35).

PH edema is directly toxic to neurons as it disrupts the blood-brain barrier (36). The blood-brain barrier primarily consists of endothelial cells with specialized tight junctions lining the blood vessels, astrocytic end-feet surrounding the blood vessels, and pericytes embedded in the basement membranes between the endothelial cells and the astrocytes. This dynamic structure is highly regulated by interactions between its cellular and ECM components (including collagen, laminin, fibronectin, hyaluronan and proteoglycan) along with integrin receptors (37,38). In the present study, the ECM-receptor interaction pathway was also identified and different ECM fibrous proteins [COL1A2, LAMB1, syndecan 2 and fibronectin 1] and integrins (ITGA5) were downregulated.

In conclusion, the present study provides several potential targets to decrease hematoma expansion and alleviate neuronal cell death following spontaneous intracerebral hemorrhage. However, further studies are required to validate this evidence and determine its clinical utility.

## Figures and Tables

**Table I:** Demographic and tissue sampling data.

Case	Sex	Age	Time S-E	Time E-T	PH	CG	CW
27	F	72	12 h	5 h	1	1	1
28	F	95	39 h	2.5 h	1	1	1
30	M	67	17 h	3 h	1	1	
31	M	86	4 days	4 h	1	1	1

F, female; M, male; S-E, stroke to exitus; E-T, exitus to tissue samples; PH, perihematomal areas; CG, contralateral grey matter; CW, contralateral white matter.

**Table II:** Enriched Gene Ontology terms of differentially expressed genes in two interaction networks.

A, PH vs. CG			

Term	Function	FDR	Genes
GO:0006816	Calcium ion transport	2.48E-04	SLC8A2, CACNA1I, CACNB2, CACNG3, CACNA2D3, ITPR1, PRKCB, TPT1, RYR2, CACNA1E, PPP3CA, TRPV6, CAMK2A, JPH1
GO:0006811	Ion transport	3.60E-04	ATP6V0E1, SCN3B, CACNB2, FXYD7, KCNMB2, KCNQ5, KCNS1, SLC1A6, ATP5S, TPT1, TRPV6, PPP3CA, SLC30A3, JPH1, CAMK2A, GABRA2, SLC8A2, KCNB1, CACNA1I, GRIA3, CACNG3, CACNA2D3, ANKH, KCNV1, ITPR1, PRKCB, SLC17A7, KCNJ4, GRIA2, GRIA1, RYR2, CACNA1E
GO:0007268	Synaptic transmission	5.94E-04	DLGAP1, GABRA2, STX1A, DLGAP2, NRXN3, CACNB2, PAH, VIPR1, SLC17A7, MAPK1, KCNQ5, GRIA2, GRIA1, CHRM1, SLC1A6, CACNA1E, PPP3CA, UNC13C, HTR2A
GO:0007267	Cell-cell signaling	0.001017	NRP1, TOLLIP, CACNB2, PAH, VIPR1, KCNQ5, CRB1, SLC1A6, PPP3CA, FGF1, GABRA2, DLGAP1, STX1A, EFNB3, NRXN3, DLGAP2, EFNB2, SLC17A7, MAPK1, GRIA2, ADM, GRIA1, CHRM1, EFNA5, CACNA1E, UNC13C, HTR2A
GO:0019226	Transmission of nerve impulse	0.001418	DLGAP1, GABRA2, STX1A, DLGAP2, NRXN3, CACNB2, PAH, VIPR1, KCNMB2, SLC17A7, MAPK1, KCNQ5, GRIA2, GRIA1, CHRM1, SLC1A6, CACNA1E, PPP3CA, UNC13C, HTR2A
GO:0030001	Metal ion transport	0.001908	SLC8A2, SCN3B, KCNB1, CACNA1I, CACNB2, CACNG3, CACNA2D3, ITPR1, KCNV1, KCNMB2, PRKCB, SLC17A7, KCNQ5, KCNJ4, KCNS1, TPT1, RYR2, CACNA1E, TRPV6, PPP3CA, SLC30A3, JPH1, CAMK2A
GO:0006812	Cation transport	0.002788	ATP6V0E1, SCN3B, CACNB2, KCNMB2, KCNQ5, KCNS1, ATP5S, TPT1, SLC30A3, PPP3CA, TRPV6, JPH1, CAMK2A, SLC8A2, KCNB1, CACNA1I, CACNG3, CACNA2D3, KCNV1, ITPR1, PRKCB, SLC17A7, KCNJ4, RYR2, CACNA1E
GO:0015674	Di-, tri-valent inorganic cation transport	0.002936	SLC8A2, CACNA1I, CACNB2, CACNG3, CACNA2D3, ITPR1, PRKCB, TPT1, RYR2, CACNA1E, PPP3CA, TRPV6, CAMK2A, JPH1
GO:0030182	Neuron differentiation	0.036294	DCC, NRP1, EFNB3, NRXN3, NTN4, DPYSL5, PTPRR, SLIT2, GPR98, ARX, NTRK3, FEZF2, DLX1, ADM, BAG1, CRB1, S100B, NEUROD2, EFNA5, OLFM3
GO:0022836	Gated channel activity	0.003996	GABRA2, SCN3B, KCNB1, CACNA1I, CACNB2, CACNG3, GRIA3, CACNA2D3, ITPR1, KCNV1, KCNMB2, KCNQ5, KCNJ4, KCNS1, GRIA2, GRIA1, RYR2, CACNA1E
GO:0005216	Ion channel activity	0.005017	GABRA2, SCN3B, KCNB1, CACNA1I, CACNB2, CACNG3, GRIA3, CACNA2D3, ITPR1, KCNV1, FXYD7, KCNMB2, KCNQ5, KCNJ4, KCNS1, GRIA2, GRIA1, RYR2, CACNA1E, TRPV6
GO:0022838	Substrate specific channel activity	0.007815	GABRA2, SCN3B, KCNB1, CACNA1I, CACNB2, CACNG3, GRIA3, CACNA2D3, ITPR1, KCNV1, FXYD7, KCNMB2, KCNQ5, KCNJ4, KCNS1, GRIA2, GRIA1, RYR2, CACNA1E, TRPV6
GO:0015267	Channel activity	0.01281	GABRA2, SCN3B, KCNB1, CACNA1I, CACNB2, CACNG3, GRIA3, CACNA2D3, ITPR1, KCNV1, FXYD7, KCNMB2, KCNQ5, KCNJ4, KCNS1, GRIA2, GRIA1, RYR2, CACNA1E, TRPV6
GO:0022803	Passive transmembrane transporter activity	0.013258	GABRA2, SCN3B, KCNB1, CACNA1I, CACNB2, CACNG3, GRIA3, CACNA2D3, ITPR1, KCNV1, FXYD7, KCNMB2, KCNQ5, KCNJ4, KCNS1, GRIA2, GRIA1, RYR2, CACNA1E, TRPV6

B, PH vs. CW			

Term	Function	FDR	Genes

GO:0001568	Blood vessel development	5.290E-04	EDNRA, GPX1, EMCN, CTGF, HMOX1, DHCR7, COL1A2, QKI, ELK3, CDH5, SCG2, ANXA2
GO:0001944	Vasculature development	6.480E-04	EDNRA, GPX1, EMCN, CTGF, HMOX1, DHCR7, COL1A2, QKI, ELK3, CDH5, SCG2, ANXA2
GO:0042127	Regulation of cell proliferation	0.001	FGFR2, DLC1, TESC, KAT2B, HCLS1, PTGS1, SPHK1, CDH5, ARNT, TRIB1, MYCN, EDNRA, AZGP1, GPX1, VDR, PTGES, HMOX1, DHCR7, FABP3, GPNMB, LAMB1, KLF4, SCG2
GO:0006952	Defense response	0.002	TF, A2M, YWHAZ, IL1R1, LYZ, HLA-C, HLA-B, PXK, STAT3, GCH1, VDAC1, CD163, CD83, INHBA, HMOX1, IL1RAPL1, RAB27A, FN1, HLA-DRA, SCG2
GO:0008283	Cell proliferation	0.003	BCAT1, NANOG, MAP2K1, NASP, ZEB2, FKBP1A, HPRT1, EDNRA, GPX1, UHRF1, DAB2, NDE1, HMOX1, GAB1, CKS2
GO:0016310	Phosphorylation	0.003	FGFR2, IRAK1, TRPM6, MAP2K1, STK24, NEK1, PIK3C2B, PRKCH, STK17A, PXK, PRKX, TRIB1, RPS6KA3, MAP4K5, PLK2, SCYL2, GAB1, RELN, MERTK, PGK1, LIPE, UGP2
GO:0006793	Phosphorus metabolic process	0.003	FGFR2, NEK1, STK17A, PXK, PRKX, TRIB1, DUSP14, GAB1, IRAK1, TRPM6, MAP2K1, STK24, PIK3C2B, PRKCH, DUSP5, RPS6KA3, MAP4K5, PLK2, SCYL2, RELN, PGK1, MERTK, LIPE, UGP2, DUSP7
GO:0006796	Phosphate metabolic process	0.003	FGFR2, NEK1, STK17A, PXK, PRKX, TRIB1, DUSP14, GAB1, IRAK1, TRPM6, MAP2K1, STK24, PIK3C2B, PRKCH, DUSP5, RPS6KA3, MAP4K5, PLK2, SCYL2, RELN, PGK1, MERTK, LIPE, UGP2, DUSP7
GO:0008285	Negative regulation of cell proliferation	0.004	DLC1, TESC, KAT2B, CDH5, TRIB1, VDR, AZGP1, PTGES, HMOX1, FABP3, GPNMB, KLF4, SCG2
GO:0006468	Protein amino acid phosphorylation	0.004	FGFR2, IRAK1, TRPM6, MAP2K1, STK24, NEK1, PRKCH, STK17A, PXK, PRKX, TRIB1, RPS6KA3, MAP4K5, PLK2, SCYL2, GAB1, RELN, MERTK, LIPE
GO:0043085	Positive regulation of catalytic activity	0.005	DLC1, IRAK1, MAP2K1, SPHK1, ZEB2, PMAIP1, TPM1, EDNRA, VDR, MAP4K5, PSMD12, GSPT1, IFT57, GAB1, GCH1, RELN
GO:0032268	Regulation of cellular protein metabolic process	0.005	HSP90AB1, DLC1, A2M, HCLS1, EIF5, ZEB2, FKBP1A, EDNRA, MAP4K5, PSMD12, TIA1, GAB1, QKI, HSPB1, PPP1R15A
GO:0044093	Positive regulation of molecular function	0.006	DLC1, IRAK1, MAP2K1, SPHK1, FKBP1A, ZEB2, PMAIP1, TPM1, GCH1, EDNRA, VDR, MAP4K5, PSMD12, GSPT1, IFT57, GAB1, RELN
GO:0051174	Regulation of phosphorus metabolic process	0.007	DLC1, IRAK1, MAP2K1, HCLS1, SPHK1, ZEB2, FKBP1A, APLP2, TRIB1, EDNRA, INHBA, MAP4K5, GAB1, CKS2, RELN
GO:0019220	Regulation of phosphate metabolic process	0.007	DLC1, IRAK1, MAP2K1, HCLS1, SPHK1, ZEB2, FKBP1A, APLP2, TRIB1, EDNRA, INHBA, MAP4K5, GAB1, CKS2, RELN
GO:0042981	Regulation of apoptosis	0.007	DLC1, IRAK1, YWHAZ, PREX1, SPHK1, ACTN1, STK17A, PMAIP1, GCH1, INHBA, GPX1, VDR, GSPT1, HMOX1, TIA1, IFT57, HSPB1, CTSB, API5, RAB27A, SCG2
GO:0043067	Regulation of programmed cell death	0.007	DLC1, IRAK1, YWHAZ, PREX1, SPHK1, ACTN1, STK17A, PMAIP1, GCH1, INHBA, GPX1, VDR, GSPT1, HMOX1, TIA1, IFT57, HSPB1, CTSB, API5, RAB27A, SCG2, DLC1
GO:0010941	Regulation of cell death	0.008	IRAK1, YWHAZ, PREX1, SPHK1, ACTN1, STK17A, PMAIP1, GCH1, INHBA, GPX1, VDR, GSPT1, HMOX1, TIA1, IFT57, HSPB1, CTSB, API5, RAB27A, SCG2

FDR, false discovery rate adjusted by BH multiple test correction method; PH, perihematomal areas; CG, contralateral grey matter; CW, contralateral white matter; BH, Benjamini Hochberg; GO, Gene Ontology; DEGs, differentially expressed genes; CAMK2A; Ca^2+^/calmodulin-dependent protein kinase II α; RYR2, ryanodine receptor 2; ITPR1, inositol 1,4,5-trisphosphate receptor, type 1; LAMB1, laminin B1; CACNA1 calcium channel, voltage-dependent, T-type, α1I subunit; COL1A, collagen type 1, α2; FN1, fibronectin 1; SPHK1, sphingosine kinase-1; EFNA5, ephrin-A5.

**Table III:** Enriched pathways of differentially expressed genes in two interaction networks.

A, PH vs. CG		

Pathway	FDR	Genes
hsa04020: Calcium signaling pathway	7.320E-05	SLC8A2, ADORA2B, CACNA1I, SPHK1, HTR4, ITPR1, PRKCB, CHRM1, RYR2, CACNA1E, PPP3CA, CAMK2A, HTR2A
hsa04010: MAPK signaling pathway	3.020E-04	MEF2C, NLK, CACNA1I, PTPRR, CACNB2, CACNG3, CACNA2D3, DDIT3, PRKCB, MAPK1, DUSP16, CACNA1E, PPP3CA, FGF1, RAPGEF2
hsa04720: Long-term potentiation	0.001	MAPK1, GRIA2, GRIA1, PPP3CA, CAMK2A, ITPR1, PRKCB
hsa04080: Neuroactive ligand-receptor interaction	0.007	GABRA2, ADORA2B, GRIA2, PTH2R, THRB, GRIA1, CNR1, CHRM1, HTR4, GRIA3, VIPR1, HTR2A, SLC8A2, CACNA1I, CACNB2, CACNG3, CACNA2D3, ITPR1, PRKCB, TPT1, RYR2, CACNA1E, PPP3CA, TRPV6, CAMK2A, JPH1

B, PH vs. CW		

Pathway	FDR	Genes

hsa04512: ECM-receptor interaction	0.019	ITGA5, COL1A2, RELN, LAMB1, SDC2, FN1
hsa05330: Allograft rejection	0.028	HLA-DRB1, HLA-A, HLA-C, HLA-B, HLA-DRA
hsa04510: Focal adhesion	0.031516	MAP2K1, ITGA5, COL1A2, ACTN1, RELN, LAMB1, VASP, VCL, FN1
hsa04514: Cell adhesion molecules	0.032	HLA-DRB1, HLA-A, CNTN2, HLA-C, HLA-B, CDH5, SDC2, HLA-DRA
hsa05332: Graft-versus-host disease	0.05	HLA-DRB1, HLA-A, HLA-C, HLA-B, HLA-DRA
hsa04940: Type I diabetes mellitus	0.042	HLA-DRB1, HLA-A, HLA-C, HLA-B, HLA-DRA

FDR, false discovery rate adjusted by Benjamini-Hochberg multiple test correction method; PH, perihematomal areas; CG, contralateral grey matter; CW, contralateral white matter; CAMK2A; Ca^2+^/calmodulin-dependent protein kinase II α; RYR2, ryanodine receptor 2; ITPR1, inositol 1,4,5-trisphosphate receptor, type 1; LAMB1, laminin B1; CACNA1 calcium channel, voltage-dependent, T-type, α1I subunit; COL1A, collagen type 1, α2; FN1, fibronectin 1; SPHK1, sphingosine kinase-1.
